# Genetic Insights into Feline Parvovirus: Evaluation of Viral Evolutionary Patterns and Association between Phylogeny and Clinical Variables

**DOI:** 10.3390/v13061033

**Published:** 2021-05-30

**Authors:** Claudia Maria Tucciarone, Giovanni Franzo, Matteo Legnardi, Elena Lazzaro, Andrea Zoia, Matteo Petini, Tommaso Furlanello, Marco Caldin, Mattia Cecchinato, Michele Drigo

**Affiliations:** 1 Department of Animal Medicine, Production and Health (MAPS), University of Padua, Viale dell’Università 16, 35020 Legnaro, Italy; giovanni.franzo@unipd.it (G.F.); matteo.legnardi@gmail.com (M.L.); eelenalazzaro@gmail.com (E.L.); mattia.cecchinato@unipd.it (M.C.); michele.drigo@unipd.it (M.D.); 2 Division of Internal Medicine, San Marco Veterinary Clinic, Viale dell’Industria 3, 35030 Veggiano, Italy; zoia.andrea06@googlemail.com (A.Z.); matteo.bullpit@gmail.com (M.P.); mc@sanmarcovet.it (M.C.); 3 Laboratory of Veterinary Diagnostics, San Marco Veterinary Private Clinic, Via dell’Industria 3, 35030 Veggiano, Italy; tf@sanmarcovet.it

**Keywords:** feline panleukopenia, molecular epidemiology, phylodynamics, disease severity, canine parvovirus

## Abstract

Feline panleukopenia is a severe disease of cats caused by feline parvovirus (FPV), and marginally canine parvovirus (CPV). Despite being less rapid than CPV, FPV evolution deserves attention, especially since outbreaks of particular severity are currently reported. This apparently different virulence needs monitoring from genetic and clinical points of view. This manuscript explored FPV molecular epidemiology at both Italian and international levels and the possible association between viral phylogeny and disease severity. Sequences from clinical cases of feline panleukopenia in Italy were obtained from 2011 to 2019, and the etiological agent was characterized, distinguishing FPV from CPV. Phylogenetic and phylodynamic analyses were conducted on Italian and international sequences. Moreover, the association between the viral sequence and clinical variables was evaluated on a group of highly characterized patients. After its origin in the 1920s, FPV showed a constant population size until a more recent expansion since 2000. Few long-distance introduction events characterized FPV spreading, however, most of its evolution occurred locally. Although without a strong statistical association, several clinical variables appeared influenced by viral phylogeny, suggesting a differential virulence potentially characterizing FPV strains. These results stress the importance of the continuous study of viral evolution and its repercussions on the disease clinical aspects.

## 1. Introduction

Carnivore protoparvovirus 1 is a recently defined species, which groups several well-known viruses as canine parvovirus (CPV) and feline parvovirus (FPV) [1], among others. These two viruses share close antigenic, phylogenetic, and evolutive relationships and cause gastroenteric disease and immunosuppression in young animals [2].

Since its panzootic emergence in the late 1970s [3], CPV has long drawn remarkable attention as one of the best characterized and most studied cases of multiple host jump. Despite its likely origin from FPV [4], CPV-2, as initially named, was able to infect only dogs and later, through a rapid evolution, new phenotypic variants were discovered that regained the capability of infecting cats [5]. FPV has remained more stable instead, both in variability and host range [6]. FPV is known since the early 20th century [7], it showed a constant viral population size and lower evolutionary rate [8] and its genetic variability was driven mainly by genetic drift [6]. Because of its features, FPV has been slightly more neglected than its conspecific CPV. 

Despite its apparently plain evolutionary pattern, FPV remains an important cause of disease in the feline population, with recurrent outbreaks especially linked to a decrease in vaccination coverage [9]. Clinical parvovirosis in cats is mainly sustained by FPV, although a small percentage (<10%) of cases is due to CPV [9]. From a clinical point of view, the manifestations of FPV and CPV in cats and dogs have been anecdotally reported to differ in the severity of the gastroenteric signs that appear worse in dogs [10], however, this difference has not been experimentally or systematically assessed in cats infected by the two viruses and the distinction of the agents remains impossible without molecular diagnostic tests.

Differently from CPV in dogs, where an association between disease severity and phylogenetic clustering has been proven [11], no data are available on the role of particular FPV strains in determining the outcome or disease severity. Few studies on feline panleukopenia have described the negative prognostic value of some factors [12,13,14], such as clinical signs (e.g., lethargy, and low body temperature) or hematologic and biochemical alterations (e.g., leukopenia, thrombocytopenia, hypoalbuminemia, and hypokalemia, low serum thyroxine concentration), whose modulation has already been associated with CPV phylogeny [11] in some cases of canine parvovirosis. Conversely, while the upsurge of feline panleukopenia cases has been tentatively ascribed to new viral types or vaccination failure due to the virus mutation, there is no consistent proof in support of these conclusions and other causes could be involved, including improper vaccination protocols or passive immunity presence [15,16]. The misdiagnosed infection of cats with CPV, potentially leading to a more severe clinical picture or diminished cross-protection [17], cannot also be excluded.

The present study was intended to explore different virus-related factors that could play a role in shaping feline panleukopenia disease. FPV macro and micro geographic distribution patterns, evolution and population dynamics were investigated, as well as the presence of CPV in cats, to depict its epidemiological relevance and a potentially different clinical impact compared to FPV. The potential association between individual strain genetic features and patients’ clinical status was also tested. The present study aimed to provide a comprehensive picture of the association between viral features, epidemiology, and host response.

## 2. Materials and Methods

Archival samples from clinical cases of confirmed parvovirosis in cats diagnosed by real-time PCR [18] were obtained from the Laboratory of the San Marco Veterinary Clinic (Veggiano, Italy). Specimens, stored at −20 °C, were EDTA whole blood, serum, bone marrow, feces, or fecal swabs, collected on the day of admission to the hospital. The complete clinical features and laboratory analysis records were extracted and associated with each sample.

DNA was extracted with DNeasy Blood and Tissue (Qiagen, Hilden, Germany) following the manufacturer’s instructions and stored at −20 °C. FPV amplification was performed on the VP1 region by PCR [19] and the specificity of the amplicons was assessed by agar gel electrophoresis. Amplicons were purified using CleanSweep™ PCR Purification Reagent kit (Applied Biosystems™, Foster City, CA, USA) and then Sanger sequenced in four overlapping reads to obtain the whole VP2 region as previously described [20]. Chromatograms were inspected for quality evaluation with FinchTV (Geospiza Inc., Seattle, WA, USA) and assembled in consensus sequences using ChromasPro 2.1.8 (Technelysium Pty Ltd, Helensvale, QLD, Australia). Full VP2 sequences were aligned to reference sequences (KC473946, FPV; EU659116, CPV) using the MUSCLE algorithm [21] implemented in MEGA X [22] and trimmed to maintain the 1755 nt-long VP2 coding region only.

For phylogenetic analyses, available FPV sequences of the VP2 gene were downloaded from Genbank and organized in an international dataset, if correctly annotated with host, date, and place of collection. The selected sequences were trimmed and aligned to those obtained in the present study using MEGA X [22]. Recombination occurrence and recombinant strain removal were performed using RDP5 [23], selecting RDP, GENECONV, Chimaera, and 3Seq for the primary scan, while the whole set of available methods was used for recombination confirmation. The method settings were adjusted based on the dataset features and recombination events were accepted only if detected by more than two methods with a significance level of *p* < 0.001 with Bonferroni correction. The presence of residual recombination signal was evaluated using GARD [24].

### 2.1. Phylogenetic Analysis, Viral Evolution and Phylogeography

A maximum likelihood (ML) phylogenetic tree was reconstructed using PhyML [25] based on the Italian sequence alignment to evaluate the genetic features and relationships among FPV strains. The substitution model was selected based on the Bayesian Information Criterion (BIC) calculated with JmodelTest [26]. The robustness of the inferred clades was assessed using the fast non-parametric version of the aLRT (Shimodaira–Hasegawa [SH]-aLRT), implemented in PhyML.

Substitution rate, time to the most recent common ancestor (TMRCA) and population dynamics were jointly estimated following the serial coalescent-based method implemented in BEAST 1.8.4 [27] on the alignment of sequences obtained from this study and international sequences. Nucleotide substitution and molecular clock models were respectively selected based on the Bayesian Information Criterion (BIC) value, computed with JmodelTest [26], and the Bayesian Factor (BF) calculated based on the marginal likelihood estimation using path sampling (PS) and stepping stones (SS) [28].

The Bayesian Skygrid method was chosen to describe the population dynamics [29], allowing the estimation of relative genetic diversity (Ne × t; i.e., the product of the Effective population size (Ne) and the generation time (t)) over time [30]. Viral migration over time among provinces (Study dataset) or countries (International dataset) was reconstructed using the discrete-trait phylogeographic approach described by Lemey et al. (2009) [31]. Population parameters and trees were sampled every 10,000 steps of a 100 million Markov Chain Monte Carlo (MCMC) run.

Convergence, mixing and Estimated Sample Size (ESS) were evaluated with Tracer1.7 software [32], and the parameter estimation was summarized as mean, median, and 95% Highest Posterior Density (95HPD), after the exclusion of an initial burn-in of 20% of the run. A maximum clade credibility tree was reconstructed using Treeannotator software [33]. The presence of statistically supported migration paths among locations was assessed through BF calculation using SpreaD3 [34]. The significance level was set to BF > 5.

### 2.2. Association between Clinicopathological Data and Italian FPV Strain Phylogenesis

Clinical and hemato-biochemical continuous variables were matched to the tips of the ML phylogenetic tree based on the sequences obtained in the present study using the phylobase R library [35]. Phylogenetic autocorrelation analysis was performed to preliminary assess if the host clinical parameters were not independent of the respective strain phylogeny. Particularly, since the focus of the present study was to assess the presence of cluster-related virulence features, the focus was restricted to the so-called “global structures” (i.e., the presence of a higher similarity in related taxa than expected by chance). A phylogenetic Principal Component Analysis (pPCA) was performed on centered and scaled variables using adephylo R package [36]. The phylogenetic proximity matrix was calculated using Abouheif’s method. This approach allowed to summarize several traits in a lower number of variables (i.e., Principal Components (PC)) exhibiting global structure. The first global component was selected and its loadings evaluated to qualitatively assess how each trait contributed to the PC. Variables with loading values in the higher quartile (i.e., most contributing variables) were selected and the presence of phylogenetic signal was evaluated for each of these variables computing the Abouheif’s Cmean using the phyloSignal package [37]. The statistical significance of the obtained index was assessed by comparison with a null hypothesis distribution (i.e., absence of phylogenetic signal) obtained performing 1000 repetitions of the test with label randomization. The significance level was set at *p*-value < 0.05 and the multiple comparisons were accounted for using the Benjamini–Hochberg False Discovery Rate (FDR) method [38].

## 3. Results

### 3.1. Datasets

During the period 2011–2019, 110 cases of parvovirosis in cats were registered at San Marco Veterinary Clinic. Biological samples were stored in the archives of the Clinic for 82 cases out of 110. The most represented matrices were feces (30), fecal swabs (22), EDTA blood (21), and serum (8), plus a bone marrow sample. After DNA extraction, 24 samples were PCR negative, likely due to DNA degradation or low viral titres. From the 58 remaining samples, 55 high quality sequences were obtained (GenBank accession numbers MW847155–MW847209).

Two CPV strains (2/55, 3.6%) were identified and classified as antigenic variant 2a. The sequences were removed from the dataset, since a meaningful statistical comparison would not have been possible due to their low number. Only the association of FPV sequences (n. 53) with the clinicopathologic information was thus analyzed, although no information was available for two samples with the remaining 51 records.

In addition to those obtained in the present study, the international dataset included 428 FPV sequences obtained from strains collected from different hosts and countries in the period 1964–2019 (Table S1).

### 3.2. Phylogenetic Analysis, Viral Evolution, and Phylogeography

**Figure 1:**
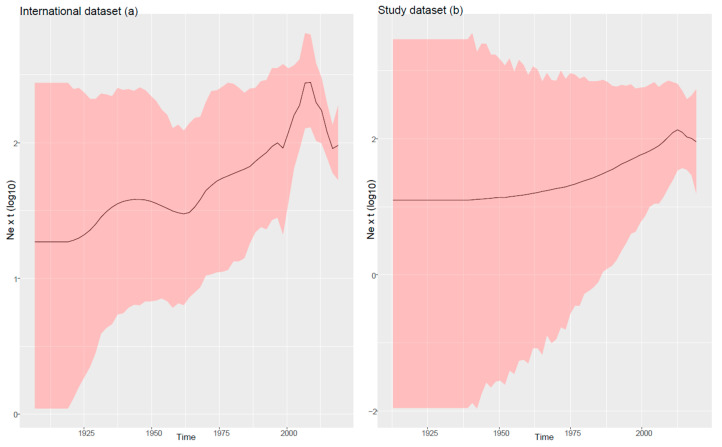
Mean relative genetic diversity (Ne x t) of FPV populations estimated on International (**a**) and Study (**b**) datasets over time. Mean, upper and lower 95HPD values are displayed.

The analysis of the international dataset estimated a mean substitution rate of 2.35 × 10^−4^ [1.70 × 10^−4^ – 3.03 × 10^−4^] for FPV and its origin in the 1920s (mean = 1927.04; 95HPD 1910.48–1934.99). After the predicted origin, FPV relative genetic diversity grew slowly until the end of the 1960s, then an increase in the viral population size was more evident until 2000, when FPV showed a rapid rise up until 2010 and followed by a reduction (Figure 1a). 

**Figure 2:**
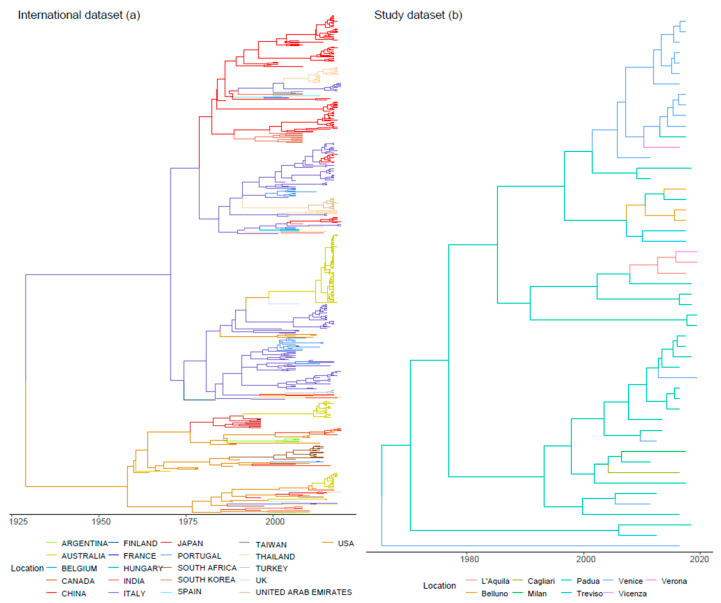
Maximum clade credibility tree phylogenetic trees reconstructed on the International (**a**) and Study (**b**) datasets. The geographical origin of the sequences (i.e., collection country 2a; Italian province 2b) is color-coded.

**Figure 3:**
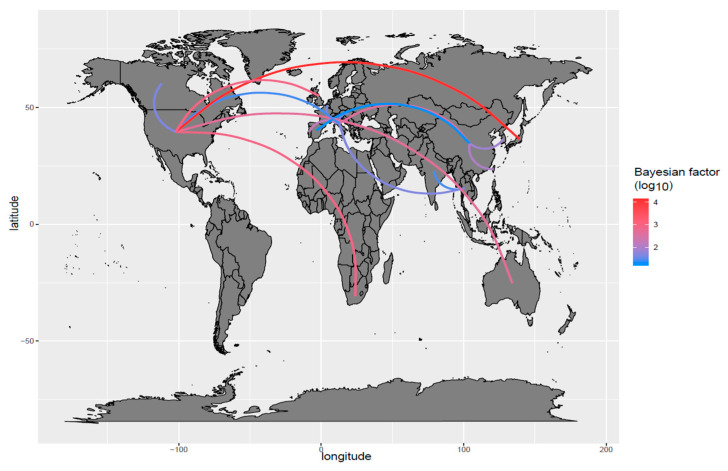
FPV migration paths. Well supported migration paths (i.e., BF > 5) among countries are depicted. The edge color is proportional to the base-10 logarithm of the migration rate. The location of each country has been matched with its centroid.

The phylogenetic tree revealed a general clustering of the sequences based on the geographic location and collection date (Figure 2a, Figure S1). Some statistically significant migration routes were proven between continents (between Italy and Belgium, Italy and China, Italy and Hungary, Portugal and Italy, South Korea and China, Spain and China, Taiwan and China, Thailand and India, Thailand and Italy, USA and Australia, USA and South Africa, USA and Italy, USA and Japan, USA and Canada, USA and UK) (Figure 3).

TMRCA for FPV in Italy was estimated in 1964 (95HPD 1912.4-2000.5). Since then, the population dynamics of Italian FPV showed slow and steady growth, slightly more pronounced after 2000 and with a minor peak around 2010 (Figure 1b). However, the broad confidence intervals weaken the value of this trend. The joint evaluation of the Italian and international dataset results suggests that Italian FPV variability originated from a few independent introduction events followed by a limited spreading. Italian sequences exhibited, in fact, a certain geographic structure and related sequences were mainly collected in the same or nearby provinces (Figure 2b). Accordingly, few migration routes emerged as statistically significant on the Italian territory (between the province pairs of L’Aquila and Padua, L’Aquila and Vicenza, Padua and Belluno, Padua and Cagliari; Padua and Venice, Padua and Treviso, Venice and Verona) (Figure S2).

### 3.3. Association between Clinicopathological Data and Italian FPV Strain Phylogenesis

**Figure 4:**
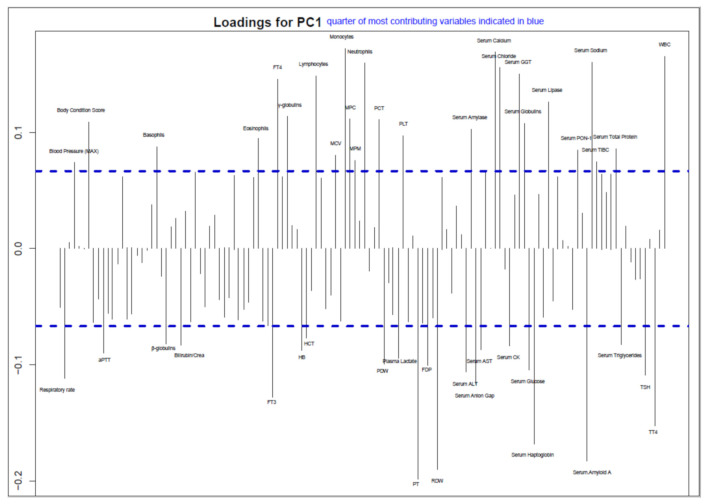
Loadings of the first global component. Only labels of variables selected for association analysis with Abouheif’s test (i.e., variables with loadings in the higher 75 percentile represented by blue dashed lines) are displayed.

The first principal component axis of pPCA explained 14% of the variability. PC1 loading evaluation showed the primary contribution of 47 out of 137 variables (Table S2), related to physical examination, complete blood count, coagulation, serum and acute phase proteins, metabolic indicators and thyroid profile (Figure 4, Table S3). However, Abouheif’s test performed on the most contributing variables to the first global axis failed to demonstrate a significant phylogenetic signal for any variable at the set significance level after controlling for multiple comparisons.

## 4. Discussion

Compared to CPV, FPV has been historically considered a more predictable pathogen for its endemic nature [8]. Since its estimated origin at the beginning of the 20th century, FPV demonstrated a relatively constant viral population size, slightly increasing over time until recent years, when a sharp decrease was observed (Figure 1a). The reasons behind this dynamic are hard to explain, however, the progressive viral population rise could be attributed to the expansion of its host population. In fact, a profound reshaping of the cat population took place in the 1900s. During the World Wars, the resident animal populations were decimated [39], and thereafter, a steady increase was evidenced, especially in Europe, in the last decades [40]. Additionally, the human migrations and travels that featured the 20th century further magnified this phenomenon by deploying both pets and their pathogens [41], generating bigger and more interconnected populations as suggested for other feline infectious diseases [42,43,44]. On the contrary, a more effective control approach or the rising owners’ awareness could be involved in the currently observed decreasing trend. It must be stressed that a sampling bias, essentially due to the limited number of available recent studies, could also explain the observed patterns and artificially decrease the actually sampled viral population and geographical niches, leading to spurious patterns.

Despite FPV worldwide distribution, a relevant geographical clustering was observed, suggestive of a relatively limited spreading capability (Figure 2a). In fact, the presence of FPV can be explained by single or few introduction events for most countries (Figure 3), followed by local circulation end evolution, similarly to what was previously described for CPV [20] and for other feline pathogens [41,42]. The acute nature of FPV infection, the severity of induced clinical signs and the lower mobility of cats compared to other pets and livestock likely limit the introduction of new strains over long distances, promoting a scenario of local evolution instead, as also proposed for other viruses [45]. Even so, owners travel more and more often with their pets, shaping the epidemiological scenario of feline diseases as well [41], and fomites also can be easily assumed to contribute to viral dispersal because of FPV remarkable environmental resistance. Still, the limited and biased sequence availability could have once again influenced the observed situation. In fact, the countries hosting multiple introduction events were also those with the higher sequence availability, thus more intensive epidemiological studies could reveal previously concealed viral spreading patterns and circulation. Interestingly, the Italian scenario well conformed with the international one, and some analogies emerged in FPV distribution and epidemiology since a province-based clustering tendency was observed (Figure 2b, Supplementary Figure S1). However, the sample collection was tied to a unique source of clinical cases to standardize the associated clinicopathological information, and the clinical records reflected the catchment area of the Clinic, unavoidably limiting the geographic depiction of viral circulation in Italy. Despite these limitations, some well-supported migration routes were also evidenced in the Italian scenario, highlighting the viral flow among provinces (Supplementary Figure S1), similarly to what was previously demonstrated for CPV [20].

Although FPV molecular epidemiology can be partially predictable, clinical parvovirosis is certainly far from uneventful. The duplicity of agents causing feline panleukopenia increases risks and reservoirs, and it could also be responsible for a different modulation of the disease. Feline panleukopenia diagnosis is often achieved by point-of-care antigen tests or PCRs specific for Carnivore protoparvoviruses, which do not usually differentiate FPV from CPV, potentially leading to an underestimation of the real contribution of the latter. Many studies reported CPV occurrence in cats [9], despite considering it to play a marginal role, while other studies suggested its lack of pathogenicity [46]. This evidence encouraged the exploration of a possible modulation of clinical alterations promoted by CPV in cats. On the other hand, only two CPV sequences belonging to the antigenic variant 2a were detected in the present study, confirming its low prevalence in cats, at least in the studied area. Furthermore, this prevented any meaningful inference on the clinical impact of this virus compared to FPV, but additional proof of the pathogenic role of CPV in cats was obtained since the enrolled animals displayed overt clinical signs in the absence of other detected pathogens.

The acquisition of a considerable number of sequences from clinically characterized cats allowed a preliminary evaluation of the association between FPV genetics and disease severity. A general trend of association between the virus phylogeny and clinical variables emerged from the analyses (Figure 4). As for CPV [11], the variables mostly contributing to the first global component were related to complete blood count (CBC), coagulation, inflammation markers, and indicators of metabolic distress. Many of these alterations have been previously identified as prognostic factors at hospital admission, such as leukocyte and thrombocyte count, serum albumin and potassium concentration [13]. However, when the phylogenetic signal was formally assessed, no variable was proven statistically significant after accounting for FDR, differently from what was observed for CPV [11], where an association emerged with an even smaller dataset. The higher similarity among FPV sequences likely reduced the phylogenetic signal and hindered the detection of potential underlying associations. Nevertheless, some variables were statistically associated with viral phylogeny when considered individually. Among these, total thyroxine (tT4) and free thyroxine (fT4) have a potentially strong biological link to the disease status since serum tT4 concentration at admission was recognized as the only prognostic value of survival by previous analyses [14]. In non-thyroidal ill patients, tT4 is considered a general indicator of sickness that decreases proportionally to the disease worsening [47]. In this case, it could be speculated that tT4 levels at admission mirror the disease severity, which could have been conditioned by the particular virulence of the strain involved, as explained by the relationship between the clinical variable and viral phylogeny. Conversely, a similar study focusing on CPV [11] did not detect a contribution of thyroid hormones to the pPCA, but of the leukocytes and neutrophilic granulocytes instead.

Even so, clinicopathological alterations are the result of complex interactions between host and pathogen, where host status, preexisting medical conditions and/or therapeutic interventions can greatly affect the virus replication or clinical picture. Retrospective studies of clinical cases cannot control for the patients’ conditions and a lack of standardization of the admission time after infection and symptom onset can further complicate the interpretation of a possible association between host manifestations and viral phylogeny. Still, this approach appears promising and should be reevaluated using a richer dataset both in sequences and relative clinical cases. In general, a deeper knowledge of FPV genetic aspects could help to monitor its epidemiological and evolutionary patterns, keeping a close eye on the acquisition of new features that could modify its virulence or impair disease control.

## Data Availability

Sequences are available in Genbank database (GenBank accession numbers MW847155–MW847209).

## Supplementary Materials

The following are available online at https://www.mdpi.com/article/10.3390/v13061033/s1, Figure S1: Phylogenetic tree reconstructed on the International dataset indicating branch support; Figure S2: FPV migration paths in Italy; Table S1 Accession numbers of sequences in the international database, Table S2 List of clinical variables considered in the study, Table S3 Summary of variable statistical association with phylogenetic structure.
