# Supplementary material for: Genetic Insights into Feline Parvovirus: Evaluation of Viral Evolutionary Patterns and Association between Phylogeny and Clinical Variables
Source: Viruses. 2021 May 30;13(6):1033. doi: 10.3390/v13061033 (PMC8230023; doi:10.3390/v13061033)
Supplement: Supplementary file 1 [file viruses-13-01033-s001.zip › viruses-1191040-supplementary/Supplementary table 1.pdf]

ACC. NUM.	STRAIN	HOST	COLLECTION PLACE	DATE
**AB000050.1**	FPV	*Felis catus*	JAPAN	1996
**AB000052.1**	FPV	*Felis catus*	JAPAN	1996
**AB000054.1**	FPV	*Felis catus*	JAPAN	1996
**AB000056.1**	FPV	*Felis catus*	JAPAN	1996
**AB000059.1**	FPV	*Felis catus*	JAPAN	1996
**AB000061.1**	FPV	*Felis catus*	JAPAN	1996
**AB000064.1**	FPV	*Felis catus*	JAPAN	1996
**AB000066.1**	FPV	*Felis catus*	JAPAN	1996
**AB000068.1**	FPV	*Felis catus*	JAPAN	1996
**AB000070.1**	FPV	*Felis catus*	JAPAN	1996
**AB054225.1**	FPV	*Felis catus*	JAPAN	2001
**AB054226.1**	FPV	*Felis catus*	JAPAN	2001
**AB054227.1**	FPV	*Felis catus*	JAPAN	2001
**AB262659.1**	FPV	*Felis catus*	JAPAN	2006
**AJ249556.1**	FPV	*Felis catus*	SOUTH AFRICA	1997
**AY606131.1**	FPV	*Felis catus*	FRANCE	2004
**D88286.1**	FPV	*Felis catus*	JAPAN	1996
**D88287.1**	FPV	*Felis catus*	JAPAN	1996
**EU221278.1**	FPV	*Felis catus*	PORTUGAL	2005
**EU221279.1**	FPV	*Felis catus*	PORTUGAL	2005
**EU221280.1**	FPV	*Felis catus*	PORTUGAL	2006
**EU221281.1**	FPV	*Felis catus*	PORTUGAL	2006
**EU360958.1**	FPV	*Felis catus*	HUNGARY	2007
**EU360959.1**	FPV	*Felis catus*	HUNGARY	2007
**EU498682.1**	FPV	*Felis catus*	ITALY	2001
**EU498683.1**	FPV	*Felis catus*	ITALY	2002
**EU498684.1**	FPV	*Felis catus*	ITALY	2002
**EU498685.1**	FPV	*Felis catus*	ITALY	2003
**EU498686.1**	FPV	*Felis catus*	ITALY	2003
**EU498687.1**	FPV	*Felis catus*	ITALY	2003
**EU498688.1**	FPV	*Felis catus*	ITALY	2004
**EU498689.1**	FPV	*Felis catus*	ITALY	2004
**EU498690.1**	FPV	*Felis catus*	ITALY	2004
**EU498691.1**	FPV	*Felis catus*	ITALY	2004
**EU498692.1**	FPV	*Felis catus*	ITALY	2004
**EU498693.1**	FPV	*Felis catus*	ITALY	2004
**EU498694.1**	FPV	*Felis catus*	ITALY	2005
**EU498695.1**	FPV	*Felis catus*	ITALY	2005
**EU498696.1**	FPV	*Felis catus*	ITALY	2006
**EU498697.1**	FPV	*Felis catus*	ITALY	2006
**EU498698.1**	FPV	*Felis catus*	ITALY	2006
**EU498699.1**	FPV	*Felis catus*	ITALY	2006
**EU498700.1**	FPV	*Felis catus*	ITALY	2006
**EU498701.1**	FPV	*Felis catus*	ITALY	2006
**EU498702.1**	FPV	*Felis catus*	ITALY	2006
**EU498703.1**	FPV	*Felis catus*	ITALY	2006
**EU498704.1**	FPV	*Felis catus*	ITALY	2006
**EU498705.1**	FPV	*Felis catus*	ITALY	2006
**EU498706.1**	FPV	*Felis catus*	ITALY	2006
**EU498707.1**	FPV	*Felis catus*	ITALY	2006
**EU498708.1**	FPV	*Felis catus*	ITALY	2006
**EU498709.1**	FPV	*Felis catus*	ITALY	2006
**EU498710.1**	FPV	*Felis catus*	ITALY	2006
**EU498711.1**	FPV	*Felis catus*	ITALY	2006
**EU498712.1**	FPV	*Felis catus*	ITALY	2006
**EU498713.1**	FPV	*Felis catus*	UK	2006

**EU498714.1**	FPV	*Felis catus*	UK	2006
**EU498715.1**	FPV	*Felis catus*	ITALY	2006
**EU498716.1**	FPV	*Felis catus*	UK	2007
**EU498717.1**	FPV	*Felis catus*	UK	2007
**EU498718.1**	FPV	*Felis catus*	ITALY	2007
**EU498719.1**	FPV	*Felis catus*	UK	2007
**EU498720.1**	FPV	*Felis catus*	ITALY	2007
**EU659111.1**	FPV	*Felis catus*	USA	1967
**EU659112.1**	FPV	*Felis catus*	USA	1964
**EU659115.1**	FPV	*Felis catus*	USA	2006
**FJ440711.1**	FPV	*Felis catus*	ARGENTINA	2007
**FJ440712.1**	FPV	*Felis catus*	ARGENTINA	2007
**FJ440713.1**	FPV	*Felis catus*	ARGENTINA	2007
**FJ440714.1**	FPV	*Felis catus*	ARGENTINA	2007
**FJ936171.1**	FPV	*Felis catus*	CHINA	2008
**HQ184189.1**	FPV	*Felis catus*	SOUTH KOREA	2008
**HQ184190.1**	FPV	*Felis catus*	SOUTH KOREA	2008
**HQ184191.1**	FPV	*Felis catus*	SOUTH KOREA	2008
**HQ184192.1**	FPV	*Felis catus*	SOUTH KOREA	2008
**HQ184193.1**	FPV	*Felis catus*	SOUTH KOREA	2008
**HQ184194.1**	FPV	*Felis catus*	SOUTH KOREA	2008
**HQ184195.1**	FPV	*Felis catus*	SOUTH KOREA	2008
**HQ184196.1**	FPV	*Felis catus*	SOUTH KOREA	2008
**HQ184197.1**	FPV	*Felis catus*	SOUTH KOREA	2008
**HQ184198.1**	FPV	*Felis catus*	SOUTH KOREA	2008
**HQ184199.1**	FPV	*Felis catus*	SOUTH KOREA	2008
**HQ184200.1**	FPV	*Felis catus*	SOUTH KOREA	2008
**HQ184201.1**	FPV	*Felis catus*	SOUTH KOREA	2008
**HQ184202.1**	FPV	*Felis catus*	SOUTH KOREA	2008
**HQ184203.1**	FPV	*Felis catus*	SOUTH KOREA	2008
**HQ184204.1**	FPV	*Felis catus*	SOUTH KOREA	2008
**JX048608.1**	FPV	*Felis catus*	TAIWAN	2011
**KC473946.1**	FPV	*Felis catus*	CHINA	2012
**KC814179.1**	FPV	*Felis catus*	CHINA	2011
**KP090139.1**	FPV	*Felis catus*	INDIA	2014
**KP280068.1**	FPV	*Felis catus*	CHINA	2014
**KP769859.1**	FPV	*Felis catus*	BELGIUM	2013
**KT240128.1**	FPV	*Felis catus*	PORTUGAL	2006
**KT240129.1**	FPV	*Felis catus*	PORTUGAL	2007
**KT240130.1**	FPV	*Felis catus*	PORTUGAL	2008
**KT240131.1**	FPV	*Felis catus*	PORTUGAL	2008
**KT240132.1**	FPV	*Felis catus*	PORTUGAL	2012
**KT240133.1**	FPV	*Felis catus*	PORTUGAL	2013
**KT240134.1**	FPV	*Felis catus*	PORTUGAL	2013
**KT240135.1**	FPV	*Felis catus*	PORTUGAL	2014
**KT240136.1**	FPV	*Felis catus*	PORTUGAL	2014
**KT357494.1**	FPV	*Felis catus*	THAILAND	2014
**KU248456.1**	FPV	*Felis catus*	PORTUGAL	2006
**KU248457.1**	FPV	*Felis catus*	PORTUGAL	2006
**KU248458.1**	FPV	*Felis catus*	PORTUGAL	2006
**KU248459.1**	FPV	*Felis catus*	PORTUGAL	2006
**KU248460.1**	FPV	*Felis catus*	PORTUGAL	2006
**KU248461.1**	FPV	*Felis catus*	PORTUGAL	2006
**KU248462.1**	FPV	*Felis catus*	PORTUGAL	2007
**KU248463.1**	FPV	*Felis catus*	PORTUGAL	2008
**KU248464.1**	FPV	*Felis catus*	PORTUGAL	2014
**KX434461**	FPV	*Felis catus*	ITALY	2015
**KX434461.1**	FPV	*Felis catus*	ITALY	2015

**KX434462**	FPV	*Felis catus*	ITALY	2015
**KX434462.1**	FPV	*Felis catus*	ITALY	2015
**KX943310.1**	FPV	*Felis catus*	ITALY	2015
**KX943311.1**	FPV	*Felis catus*	ITALY	2015
**KX943312.1**	FPV	*Felis catus*	ITALY	2015
**KX943313.1**	FPV	*Felis catus*	ITALY	2015
**KX943314.1**	FPV	*Felis catus*	ITALY	2015
**KX943315.1**	FPV	*Felis catus*	ITALY	2015
**KX943316.1**	FPV	*Felis catus*	ITALY	2015
**KX943317.1**	FPV	*Felis catus*	ITALY	2015
**KX943318.1**	FPV	*Felis catus*	ITALY	2015
**KY451727.1**	FPV	*Felis catus*	CHINA	2016
**M24002.1**	FPV	*Felis catus*	USA	1988
**M24004.1**	FPV	*Felis catus*	USA	1988
**MF541119.1**	FPV	*Felis catus*	CHINA	2016
**MF541120.1**	FPV	*Felis catus*	CHINA	2016
**MF541121.1**	FPV	*Felis catus*	CHINA	2016
**MF541122.1**	FPV	*Felis catus*	CHINA	2016
**MF541123.1**	FPV	*Felis catus*	CHINA	2017
**MF541124.1**	FPV	*Felis catus*	CHINA	2017
**MF541125.1**	FPV	*Felis catus*	CHINA	2016
**MF541126.1**	FPV	*Felis catus*	CHINA	2017
**MF541127.1**	FPV	*Felis catus*	CHINA	2017
**MF541128.1**	FPV	*Felis catus*	CHINA	2017
**MF541129.1**	FPV	*Felis catus*	CHINA	2017
**MF541130.1**	FPV	*Felis catus*	CHINA	2017
**MF541131.1**	FPV	*Felis catus*	CHINA	2017
**MF541132.1**	FPV	*Felis catus*	CHINA	2017
**MF541133.1**	FPV	*Felis catus*	CHINA	2017
**MF541134.1**	FPV	*Felis catus*	CHINA	2017
**MF541135.1**	FPV	*Felis catus*	CHINA	2017
**MF541136.1**	FPV	*Felis catus*	CHINA	2017
**MF541137.1**	FPV	*Felis catus*	CHINA	2017
**MF541138.1**	FPV	*Felis catus*	CHINA	2017
**MF541139.1**	FPV	*Felis catus*	CHINA	2017
**MF541140.1**	FPV	*Felis catus*	CHINA	2016
**MH165481.1**	FPV	*Felis catus*	CHINA	2015
**MH165482.1**	FPV	*Felis catus*	CHINA	2014
**MH329286.1**	FPV	*Felis catus*	CHINA	2016
**MH496766.1**	FPV	*Felis catus*	TURKEY	2017
**MH496767.1**	FPV	*Felis catus*	TURKEY	2017
**MH496768.1**	FPV	*Felis catus*	TURKEY	2017
**MH496769.1**	FPV	*Felis catus*	TURKEY	2017
**MH496770.1**	FPV	*Felis catus*	TURKEY	2017
**MH559110.1**	FPV	*Felis catus*	INDIA	2018
**MK266782.1**	FPV	*Felis catus*	CHINA	2017
**MK266783.1**	FPV	*Felis catus*	CHINA	2018
**MK266784.1**	FPV	*Felis catus*	CHINA	2017
**MK266785.1**	FPV	*Felis catus*	CHINA	2017
**MK266786.1**	FPV	*Felis catus*	CHINA	2017
**MK266787.1**	FPV	*Felis catus*	CHINA	2017
**MK266788.1**	FPV	*Felis catus*	CHINA	2017
**MK266789.1**	FPV	*Felis catus*	CHINA	2017
**MK266790.1**	FPV	*Felis catus*	CHINA	2017
**MK266791.1**	FPV	*Felis catus*	CHINA	2017
**MK266792.1**	FPV	*Felis catus*	CHINA	2017
**MK266793.1**	FPV	*Felis catus*	CHINA	2018
**MK266794.1**	FPV	*Felis catus*	CHINA	2018

**MK266795.1**	FPV	*Felis catus*	CHINA	2018
**MK266796.1**	FPV	*Felis catus*	CHINA	2018
**MK266797.1**	FPV	*Felis catus*	CHINA	2018
**MK266798.1**	FPV	*Felis catus*	CHINA	2017
**MK266799.1**	FPV	*Felis catus*	CHINA	2017
**MK295775.1**	FPV	*Felis catus*	CHINA	2017
**MK413724**	FPV	*Felis catus*	ITALY	2013
**MK413724.1**	FPV	*Felis catus*	ITALY	2013
**MK413725**	FPV	*Felis catus*	ITALY	2014
**MK413725.1**	FPV	*Felis catus*	ITALY	2014
**MK413726**	FPV	*Felis catus*	ITALY	2015
**MK413726.1**	FPV	*Felis catus*	ITALY	2015
**MK413727**	FPV	*Felis catus*	ITALY	2015
**MK413727.1**	FPV	*Felis catus*	ITALY	2015
**MK413728**	FPV	*Felis catus*	ITALY	2015
**MK413728.1**	FPV	*Felis catus*	ITALY	2015
**MK413729**	FPV	*Felis catus*	ITALY	2015
**MK413729.1**	FPV	*Felis catus*	ITALY	2015
**MK413730**	FPV	*Felis catus*	ITALY	2015
**MK413730.1**	FPV	*Felis catus*	ITALY	2015
**MK413731**	FPV	*Felis catus*	ITALY	2015
**MK413731.1**	FPV	*Felis catus*	ITALY	2015
**MK413737**	FPV	*Felis catus*	ITALY	2017
**MK413737.1**	FPV	*Felis catus*	ITALY	2017
**MK413738**	FPV	*Felis catus*	ITALY	2017
**MK413738.1**	FPV	*Felis catus*	ITALY	2017
**MK425497.1**	FPV	*Felis catus*	THAILAND	2018
**MK425498.1**	FPV	*Felis catus*	THAILAND	2018
**MK425499.1**	FPV	*Felis catus*	THAILAND	2018
**MK425500.1**	FPV	*Felis catus*	THAILAND	2018
**MK425501.1**	FPV	*Felis catus*	THAILAND	2018
**MK425502.1**	FPV	*Felis catus*	THAILAND	2018
**MK425503.1**	FPV	*Felis catus*	THAILAND	2018
**MK425504.1**	FPV	*Felis catus*	THAILAND	2018
**MK425505.1**	FPV	*Felis catus*	THAILAND	2018
**MK425506.1**	FPV	*Felis catus*	THAILAND	2018
**MK425507.1**	FPV	*Felis catus*	THAILAND	2018
**MK570637.1**	FPV	*Felis catus*	AUSTRALIA	2015
**MK570638.1**	FPV	*Felis catus*	AUSTRALIA	2015
**MK570639.1**	FPV	*Felis catus*	AUSTRALIA	2015
**MK570640.1**	FPV	*Felis catus*	AUSTRALIA	2015
**MK570641.1**	FPV	*Felis catus*	AUSTRALIA	2015
**MK570642.1**	FPV	*Felis catus*	AUSTRALIA	2015
**MK570643.1**	FPV	*Felis catus*	AUSTRALIA	2015
**MK570644.1**	FPV	*Felis catus*	AUSTRALIA	2015
**MK570645.1**	FPV	*Felis catus*	AUSTRALIA	2015
**MK570646.1**	FPV	*Felis catus*	AUSTRALIA	2015
**MK570647.1**	FPV	*Felis catus*	AUSTRALIA	2015
**MK570648.1**	FPV	*Felis catus*	AUSTRALIA	2016
**MK570649.1**	FPV	*Felis catus*	AUSTRALIA	2016
**MK570650.1**	FPV	*Felis catus*	AUSTRALIA	2016
**MK570651.1**	FPV	*Felis catus*	AUSTRALIA	2016
**MK570652.1**	FPV	*Felis catus*	AUSTRALIA	2016
**MK570653.1**	FPV	*Felis catus*	AUSTRALIA	2016
**MK570654.1**	FPV	*Felis catus*	AUSTRALIA	2017
**MK570655.1**	FPV	*Felis catus*	AUSTRALIA	2017
**MK570656.1**	FPV	*Felis catus*	AUSTRALIA	2017
**MK570657.1**	FPV	*Felis catus*	AUSTRALIA	2017

**MK570658.1**	FPV	*Felis catus*	AUSTRALIA	2017
**MK570659.1**	FPV	*Felis catus*	AUSTRALIA	2017
**MK570660.1**	FPV	*Felis catus*	AUSTRALIA	2017
**MK570661.1**	FPV	*Felis catus*	AUSTRALIA	2017
**MK570662.1**	FPV	*Felis catus*	AUSTRALIA	2017
**MK570663.1**	FPV	*Felis catus*	AUSTRALIA	2017
**MK570664.1**	FPV	*Felis catus*	AUSTRALIA	2017
**MK570665.1**	FPV	*Felis catus*	AUSTRALIA	2017
**MK570666.1**	FPV	*Felis catus*	AUSTRALIA	2017
**MK570667.1**	FPV	*Felis catus*	AUSTRALIA	2017
**MK570668.1**	FPV	*Felis catus*	AUSTRALIA	2017
**MK570669.1**	FPV	*Felis catus*	AUSTRALIA	2017
**MK570670.1**	FPV	*Felis catus*	AUSTRALIA	2017
**MK570671.1**	FPV	*Felis catus*	AUSTRALIA	2017
**MK570672.1**	FPV	*Felis catus*	AUSTRALIA	2017
**MK570673.1**	FPV	*Felis catus*	AUSTRALIA	2017
**MK570674.1**	FPV	*Felis catus*	AUSTRALIA	2017
**MK570675.1**	FPV	*Felis catus*	AUSTRALIA	2018
**MK570676.1**	FPV	*Felis catus*	AUSTRALIA	2018
**MK570677.1**	FPV	*Felis catus*	AUSTRALIA	2017
**MK570678.1**	FPV	*Felis catus*	AUSTRALIA	2017
**MK570679.1**	FPV	*Felis catus*	AUSTRALIA	2017
**MK570680.1**	FPV	*Felis catus*	AUSTRALIA	2017
**MK570681.1**	FPV	*Felis catus*	AUSTRALIA	2017
**MK570682.1**	FPV	*Felis catus*	AUSTRALIA	2017
**MK570683.1**	FPV	*Felis catus*	AUSTRALIA	2017
**MK570684.1**	FPV	*Felis catus*	AUSTRALIA	2017
**MK570685.1**	FPV	*Felis catus*	AUSTRALIA	2017
**MK570686.1**	FPV	*Felis catus*	AUSTRALIA	2017
**MK570687.1**	FPV	*Felis catus*	AUSTRALIA	2017
**MK570688.1**	FPV	*Felis catus*	AUSTRALIA	2017
**MK570689.1**	FPV	*Felis catus*	AUSTRALIA	2017
**MK570690.1**	FPV	*Felis catus*	AUSTRALIA	2017
**MK570691.1**	FPV	*Felis catus*	AUSTRALIA	2017
**MK570692.1**	FPV	*Felis catus*	AUSTRALIA	2017
**MK570693.1**	FPV	*Felis catus*	AUSTRALIA	2017
**MK570694.1**	FPV	*Felis catus*	AUSTRALIA	2017
**MK570695.1**	FPV	*Felis catus*	AUSTRALIA	2017
**MK570696.1**	FPV	*Felis catus*	AUSTRALIA	2017
**MK570697.1**	FPV	*Felis catus*	AUSTRALIA	2017
**MK570698.1**	FPV	*Felis catus*	AUSTRALIA	2017
**MK570699.1**	FPV	*Felis catus*	AUSTRALIA	2017
**MK570700.1**	FPV	*Felis catus*	AUSTRALIA	2017
**MK570701.1**	FPV	*Felis catus*	AUSTRALIA	2017
**MK570702.1**	FPV	*Felis catus*	AUSTRALIA	2017
**MK570703.1**	FPV	*Felis catus*	AUSTRALIA	2017
**MK570704.1**	FPV	*Felis catus*	AUSTRALIA	2017
**MK570705.1**	FPV	*Felis catus*	AUSTRALIA	2017
**MK570706.1**	FPV	*Felis catus*	AUSTRALIA	2018
**MK570707.1**	FPV	*Felis catus*	AUSTRALIA	2018
**MK570708.1**	FPV	*Felis catus*	AUSTRALIA	2017
**MK570709.1**	FPV	*Felis catus*	AUSTRALIA	2017
**MK570710.1**	FPV	*Felis catus*	AUSTRALIA	2017
**MK570711.1**	FPV	*Felis catus*	AUSTRALIA	2018
**MK570712.1**	FPV	*Felis catus*	AUSTRALIA	2018
**MK570713.1**	FPV	*Felis catus*	AUSTRALIA	2018
**MK570714.1**	FPV	*Felis catus*	AUSTRALIA	2018
**MK570715.1**	FPV	*Felis catus*	AUSTRALIA	2018

**MK570716.1**	FPV	*Felis catus*	UNITED ARAB EMIRATES	2017
**MK570717.1**	FPV	*Felis catus*	UNITED ARAB EMIRATES	2017
**MK570718.1**	FPV	*Felis catus*	UNITED ARAB EMIRATES	2017
**MK570719.1**	FPV	*Felis catus*	UNITED ARAB EMIRATES	2017
**MK570720.1**	FPV	*Felis catus*	UNITED ARAB EMIRATES	2017
**MK570721.1**	FPV	*Felis catus*	UNITED ARAB EMIRATES	2017
**MK570722.1**	FPV	*Felis catus*	UNITED ARAB EMIRATES	2017
**MK570723.1**	FPV	*Felis catus*	UNITED ARAB EMIRATES	2017
**MK570724.1**	FPV	*Felis catus*	UNITED ARAB EMIRATES	2017
**MK570725.1**	FPV	*Felis catus*	UNITED ARAB EMIRATES	2017
**MK570726.1**	FPV	*Felis catus*	AUSTRALIA	2017
**MK570727.1**	FPV	*Felis catus*	AUSTRALIA	2017
**MK570728.1**	FPV	*Felis catus*	AUSTRALIA	2016
**MK570729.1**	FPV	*Felis catus*	AUSTRALIA	2017
**MK570730.1**	FPV	*Felis catus*	AUSTRALIA	2017
**MK570731.1**	FPV	*Felis catus*	AUSTRALIA	2017
**MK570732.1**	FPV	*Felis catus*	AUSTRALIA	2017
**MK570733.1**	FPV	*Felis catus*	AUSTRALIA	2017
**MK570734.1**	FPV	*Felis catus*	AUSTRALIA	2017
**MK570735.1**	FPV	*Felis catus*	AUSTRALIA	2017
**MK570736.1**	FPV	*Felis catus*	AUSTRALIA	2017
**MK570737.1**	FPV	*Felis catus*	AUSTRALIA	2017
**MK570738.1**	FPV	*Felis catus*	AUSTRALIA	2017
**MK570739.1**	FPV	*Felis catus*	AUSTRALIA	2016
**MK570740.1**	FPV	*Felis catus*	AUSTRALIA	2016
**MK570741.1**	FPV	*Felis catus*	AUSTRALIA	2017
**MK570742.1**	FPV	*Felis catus*	AUSTRALIA	2017
**MK570743.1**	FPV	*Felis catus*	AUSTRALIA	2017
**MK570744.1**	FPV	*Felis catus*	AUSTRALIA	2017
**MK570745.1**	FPV	*Felis catus*	AUSTRALIA	2017
**MK570746.1**	FPV	*Felis catus*	AUSTRALIA	2017
**MK570747.1**	FPV	*Felis catus*	AUSTRALIA	2017
**MK570748.1**	FPV	*Felis catus*	AUSTRALIA	2017
**MK570749.1**	FPV	*Felis catus*	AUSTRALIA	2016
**MK671150.1**	FPV	*Felis catus*	CHINA	2016
**MK671151.1**	FPV	*Felis catus*	CHINA	2016
**MK671152.1**	FPV	*Felis catus*	CHINA	2016
**MK671153.1**	FPV	*Felis catus*	CHINA	2016
**MK671154.1**	FPV	*Felis catus*	CHINA	2016
**MK671155.1**	FPV	*Felis catus*	CHINA	2016
**MK671156.1**	FPV	*Felis catus*	CHINA	2016
**MK671157.1**	FPV	*Felis catus*	CHINA	2017
**MK671158.1**	FPV	*Felis catus*	CHINA	2017
**MK671159.1**	FPV	*Felis catus*	CHINA	2017
**MK671160.1**	FPV	*Felis catus*	CHINA	2017
**MK671161.1**	FPV	*Felis catus*	CHINA	2017
**MK671162.1**	FPV	*Felis catus*	CHINA	2017
**MK671163.1**	FPV	*Felis catus*	CHINA	2017

**MK671164.1**	FPV	*Felis catus*	CHINA	2017
**MK671165.1**	FPV	*Felis catus*	CHINA	2017
**MK671166.1**	FPV	*Felis catus*	CHINA	2017
**MK671167.1**	FPV	*Felis catus*	CHINA	2017
**MK671168.1**	FPV	*Felis catus*	CHINA	2017
**MK671169.1**	FPV	*Felis catus*	CHINA	2017
**MK671170.1**	FPV	*Felis catus*	CHINA	2017
**MK671171.1**	FPV	*Felis catus*	CHINA	2017
**MK671172.1**	FPV	*Felis catus*	CHINA	2018
**MK671173.1**	FPV	*Felis catus*	CHINA	2018
**MK671174.1**	FPV	*Felis catus*	CHINA	2018
**MK671175.1**	FPV	*Felis catus*	CHINA	2018
**MK671176.1**	FPV	*Felis catus*	CHINA	2018
**MK671177.1**	FPV	*Felis catus*	CHINA	2018
**MK671178.1**	FPV	*Felis catus*	CHINA	2018
**MK671179.1**	FPV	*Felis catus*	CHINA	2018
**MK671180.1**	FPV	*Felis catus*	CHINA	2018
**MK671181.1**	FPV	*Felis catus*	CHINA	2018
**MK671182.1**	FPV	*Felis catus*	CHINA	2018
**MK671183.1**	FPV	*Felis catus*	CHINA	2018
**MK671184.1**	FPV	*Felis catus*	CHINA	2018
**MK671185.1**	FPV	*Felis catus*	CHINA	2018
**MK671186.1**	FPV	*Felis catus*	CHINA	2018
**MK671187.1**	FPV	*Felis catus*	CHINA	2018
**MK671188.1**	FPV	*Felis catus*	CHINA	2018
**MN127779.1**	FPV	*Felis catus*	THAILAND	2018
**MN127780.1**	FPV	*Felis catus*	THAILAND	2019
**MN127781.1**	FPV	*Felis catus*	THAILAND	2019
**MN603975.1**	FPV	*Felis catus*	UNITED ARAB EMIRATES	2017
**MN603976.1**	FPV	*Felis catus*	AUSTRALIA	2010
**X55115.1**	FPV	*Felis catus*	AUSTRALIA	1970
**MT274378.1**	FPV	*Meles meles*	ITALY	2019
**MT274377.1**	FPV	*Vulpes vulpes*	ITALY	2017
**MN908257.1**	FPV	*Panthera tigris*	CHINA	2019
**MN862749.1**	FPV	*Lontra canadensis*	CANADA	2019
**MN862748.1**	FPV	*Lontra canadensis*	CANADA	2019
**MN862747.1**	FPV	*Neovison vison*	CANADA	2019
**MN862746.1**	FPV	*Neovison vison*	CANADA	2017
**MN862745.1**	FPV	*Martes americana*	CANADA	2017
**MN862744.1**	FPV	*Martes americana*	CANADA	2016
**MN862743.1**	FPV	*Neovison vison*	CANADA	2018
**MN451692.1**	FPV	*Procyon lotor*	USA	2011
**MN451652.1**	FPV	*Vulpes lagopus*	FINLAND	1983
**MK982094.1**	FPV	*Panthera tigris*	CHINA	2017
**MH669800.1**	FPV	*Prionodon linsang*	THAILAND	2015
**MG764511.1**	FPV	*Panthera leo*	CHINA	2015
**MG764510.1**	FPV	*Panthera tigris*	CHINA	1999
**MF069447.1**	FPV	*Procyon lotor*	CANADA	2016
**MF069446.1**	FPV	*Procyon lotor*	CANADA	2010
**MF069445.1**	FPV	*Procyon lotor*	CANADA	2015
**KX900570.1**	FPV	*Panthera onca*	CHINA	1986
**KX685354.1**	FPV	*Panthera tigris*	CHINA	2016
**KT357493.1**	FPV	*Viverricula indica*	THAILAND	2013
**KT357492.1**	FPV	*Viverricula indica*	THAILAND	2013
**KT357491.1**	FPV	*Viverricula indica*	THAILAND	2013
**KP682526.1**	FPV	*Martes foina*	SPAIN	2002
**KP682520.1**	FPV	*Meles meles*	SPAIN	2013

**KP033250.1**	FPV	*Leptailurus serval*	SOUTH AFRICA	2014
**KP033249.1**	FPV	*Acinonyx jubatus*	SOUTH AFRICA	2014
**KP033248.1**	FPV	*Acinonyx jubatus*	SOUTH AFRICA	2014
**KP033247.1**	FPV	*Acinonyx jubatus*	SOUTH AFRICA	2014
**KP033246.1**	FPV	*Acinonyx jubatus*	SOUTH AFRICA	2014
**KP033243.1**	FPV	*Leopardus pardalis*	SOUTH AFRICA	2014
**KP033242.1**	FPV	*Leptailurus serval*	SOUTH AFRICA	2014
**KP033241.1**	FPV	*Panthera leo*	SOUTH AFRICA	2006
**KP033240.1**	FPV	*Acinonyx jubatus*	SOUTH AFRICA	2013
**KP033239.1**	FPV	*Acinonyx jubatus*	SOUTH AFRICA	2013
**KP033238.1**	FPV	*Leptailurus serval*	SOUTH AFRICA	2013
**KP033237.1**	FPV	*Acinonyx jubatus*	SOUTH AFRICA	2012
**KP033236.1**	FPV	*Acinonyx jubatus*	SOUTH AFRICA	2012
**KP033235.1**	FPV	*Acinonyx jubatus*	SOUTH AFRICA	2012
**KP033234.1**	FPV	*Acinonyx jubatus*	SOUTH AFRICA	2012
**KP019621.2**	FPV	*Viverricula indica*	THAILAND	2013
**KP019620.1**	FPV	*Viverricula indica*	THAILAND	2013
**KP019619.1**	FPV	*Viverricula indica*	THAILAND	2013
**KP019618.1**	FPV	*Viverricula indica*	THAILAND	2013
**KP019617.1**	FPV	*Viverricula indica*	THAILAND	2013
**KM624023.1**	FPV	*Procyon lotor*	USA	1978
**KJ813895.1**	FPV	*Procyon lotor*	USA	2012
**KJ813894.1**	FPV	*Procyon lotor*	USA	2012
**KJ813893.1**	FPV	*Lynx rufus*	USA	2013
**JX475270.1**	FPV	*Procyon lotor*	USA	2012
**JX475259.1**	FPV	*Puma concolor*	USA	2010
**JX475256.1**	FPV	*Puma concolor*	USA	2011
**JX475254.1**	FPV	*Puma concolor*	USA	2010
**JX475253.1**	FPV	*Puma concolor*	USA	2010
**JX475245.1**	FPV	*Puma concolor*	USA	2010
**JN867596.1**	FPV	*Procyon lotor*	USA	1978
**JN867595.1**	FPV	*Procyon lotor*	USA	1978
**JN867594.1**	FPV	*Procyon lotor*	USA	1990
**JN867593.1**	FPV	*Procyon lotor*	USA	2010
**JF422105.2**	FPV	*Herpestes ichneumon*	PORTUGAL	2009

Supplementary table 1: accession numbers of sequences in the international database.
