# Supplementary material for: Genetic Insights into Feline Parvovirus: Evaluation of Viral Evolutionary Patterns and Association between Phylogeny and Clinical Variables
Source: Viruses. 2021 May 30;13(6):1033. doi: 10.3390/v13061033 (PMC8230023; doi:10.3390/v13061033)
Supplement: Supplementary file 1 [file viruses-13-01033-s001.zip › viruses-1191040-supplementary/Supplementary table 2.pdf]

A/G ratio
ACTH
Activated partial thromboplastin time
Age
Albumin %
Aldosteron
Alpha-1-globulins
Alpha-2-globulins
Ammonia/Crea
Ammonium
Amylase/Crea
Antithrombin
Basophils
Beta-1-globulins
Beta-2-globulins
Beta-3-globulins
Beta-globulins
Bile Acids/Crea
Bilirubin/Crea
Blood Pressure (MAX)
Blood Pressure (MEAN)
Blood Pressure (MIN)
Body Condition Score
Body weight
Breed
Calcium/Crea
Calculated Osmolality
CH
CHCM
CHDW
Chloride/Crea
Cortisol
Cortisol/Creatinuria
Creatinine
Creatinuria
Death
DGGR-lipase
DHEA
Diet
EF Calcium
EF Chloride
EF Posphate
EF Potassium
EF Sodium
EF Urea
Eosinophils
FDP
Fibrinogen
FT3
FT4
Gamma-globulins
GGT/Crea
Glucose/Crea
HB
HCT
HDW
Hospitalization time
Housing
HPT
Lipase/Crea
Lymphocytes
MCHC
MCHC/CHCM
MCV
Monocytes
MPC
MPM
MPV
Neutrophils
Osmolal Gap
Osmolality
Patient status
PCDW
PCT
PDW
pH
Plasma Lactate
PLT
PMDW
Potassium/Crea
Prothrombin Time (PT)
PU/CU
Pulse rate
Quantitative FDP
RBC
RDW
Respiratory Rate
Saturation
Serum A/G
Serum Albumin
Serum ALP
Serum ALT
Serum Amylase
Serum Amyloid A
Serum Anion Gap
Serum AST
Serum BCHE
Serum Ca x Pi
Serum Calcium
Serum Chloride
Serum Cholesterol
Serum CK
Serum Creatinine
Serum GGT
Serum Globulins
Serum Glucose
Serum Haptoglobin
Serum Iron
Serum LDH
Serum Lipase
Serum Magnesium
Serum Measured Osmolality
Serum Na/K
Serum Osmolal Gap
Serum Phosphate
Serum PON-1
Serum Potassium
Serum Sodium
Serum TIBC
Serum Tonicity
Serum Total Bilirubin
Serum Total Carbon Dioxide
Serum Total Protein
Serum Triglycerides
Serum UIBC
Serum Urea
Sex
Sexual status
Sodium/Crea
Specific gravity
Temperature
Time of death
TSH
TT4
TTKG
Vaccinations
WBC

Supplementary table 2: list of clinical variables considered in the study.
