# Supplementary material for: Genetic Insights into Feline Parvovirus: Evaluation of Viral Evolutionary Patterns and Association between Phylogeny and Clinical Variables
Source: Viruses. 2021 May 30;13(6):1033. doi: 10.3390/v13061033 (PMC8230023; doi:10.3390/v13061033)
Supplement: Supplementary file 1 [file viruses-13-01033-s001.zip › viruses-1191040-supplementary/Supplementary table 3.pdf]

Clinical variable	Cmean	p-value
MPC	0.208166880926768	0.010989010989011
TT4	0.201842183251493	0.018981018981019
FT4	0.16616365927182	0.036963036963037
Serum Calcium	0.141442466269733	0.045954045954046
Serum AST	0.121647305720535	0.04995004995005
MPM	0.145456484613027	0.0559440559440559
PCT	0.120613015232448	0.0609390609390609
Serum Anion Gap	0.124232778292131	0.0679320679320679
FDP	0.117513481735115	0.0689310689310689
Serum Total Protein	0.113619556228564	0.0829170829170829
PLT	0.111289590774363	0.0889110889110889
RDW	0.115249410202735	0.102897102897103
PDW	0.0929059716450843	0.103896103896104
Prothrombin Time (PT)	0.0731140390145689	0.124875124875125
Serum Glucose	0.0731110218225893	0.134865134865135
FT3	-0.00966458976863443	0.13986013986014
Plasma Lactate	0.0815960140323062	0.144855144855145
Serum Globulins	0.0766381222289164	0.15984015984016
Serum Triglycerides	0.0401040617745541	0.162837162837163
Serum ALT	0.0365046264846605	0.193806193806194
Serum Amyloid A	0.0645363867334716	0.195804195804196
Serum Chloride	0.0637583107510727	0.205794205794206
Serum Haptoglobin	0.0617916606472676	0.21978021978022
Bilirubin/Crea	0.0457493651430743	0.228771228771229
Serum TIBC	0.0509760376788592	0.231768231768232
Gamma-globulins	0.0370884898612331	0.262737262737263
Respiratory Rate	0.0243412798066781	0.287712287712288
Lymphocytes	0.0204471483786648	0.303696303696304
WBC	0.00634463879555399	0.322677322677323
Neutrophils	-0.00447748283898707	0.33966033966034
Serum Sodium	0.0148742553188903	0.345654345654346
Serum PON-1	0.0123099778353257	0.360639360639361
Monocytes	-0.0109884335652999	0.375624375624376
Basophils	-0.02575116909475	0.411588411588412
Eosinophils	-0.0264709030102236	0.441558441558442
Beta-globulins	-0.0106573564897357	0.446553446553447
TSH	-0.0266062476745689	0.452547452547453
Serum Amylase	-0.0181001280340403	0.466533466533467
Activated Partial Thromboplastin Time (aPTT)	-0.0309818271393027	0.485514485514486
Serum Lipase	-0.032030630478711	0.522477522477522
Serum CK	-0.0372827426548352	0.55044955044955
Body Condition Score	-0.0340138129732091	0.58041958041958
Serum GGT	-0.0690284305550033	0.717282717282717
Blood Pressure (MAX)	-0.0908907600791137	0.762237762237762
HCT	-0.0826410102827217	0.77022977022977
HB	-0.122827818684888	0.862137862137862
MCV	-0.169010992616038	0.938061938061938

Supplementary table 3: summary of variable statistical association with phylogenetic structure. For each variable the calculated Cmean and the respective p-value are reported.
