# Supplementary figures and images for: Genetic Insights into Feline Parvovirus: Evaluation of Viral Evolutionary Patterns and Association between Phylogeny and Clinical Variables
Source: Viruses. 2021 May 30;13(6):1033. doi: 10.3390/v13061033 (PMC8230023; doi:10.3390/v13061033)

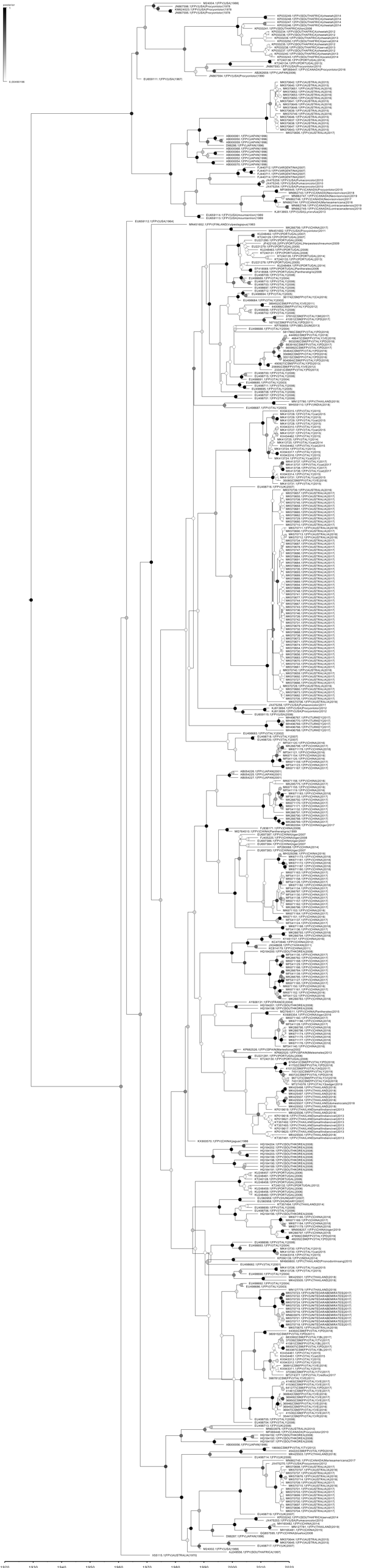

Supplement: Supplementary file 1 [file viruses-13-01033-s001.zip › viruses-1191040-supplementary/Supplementary figure 1.pdf]

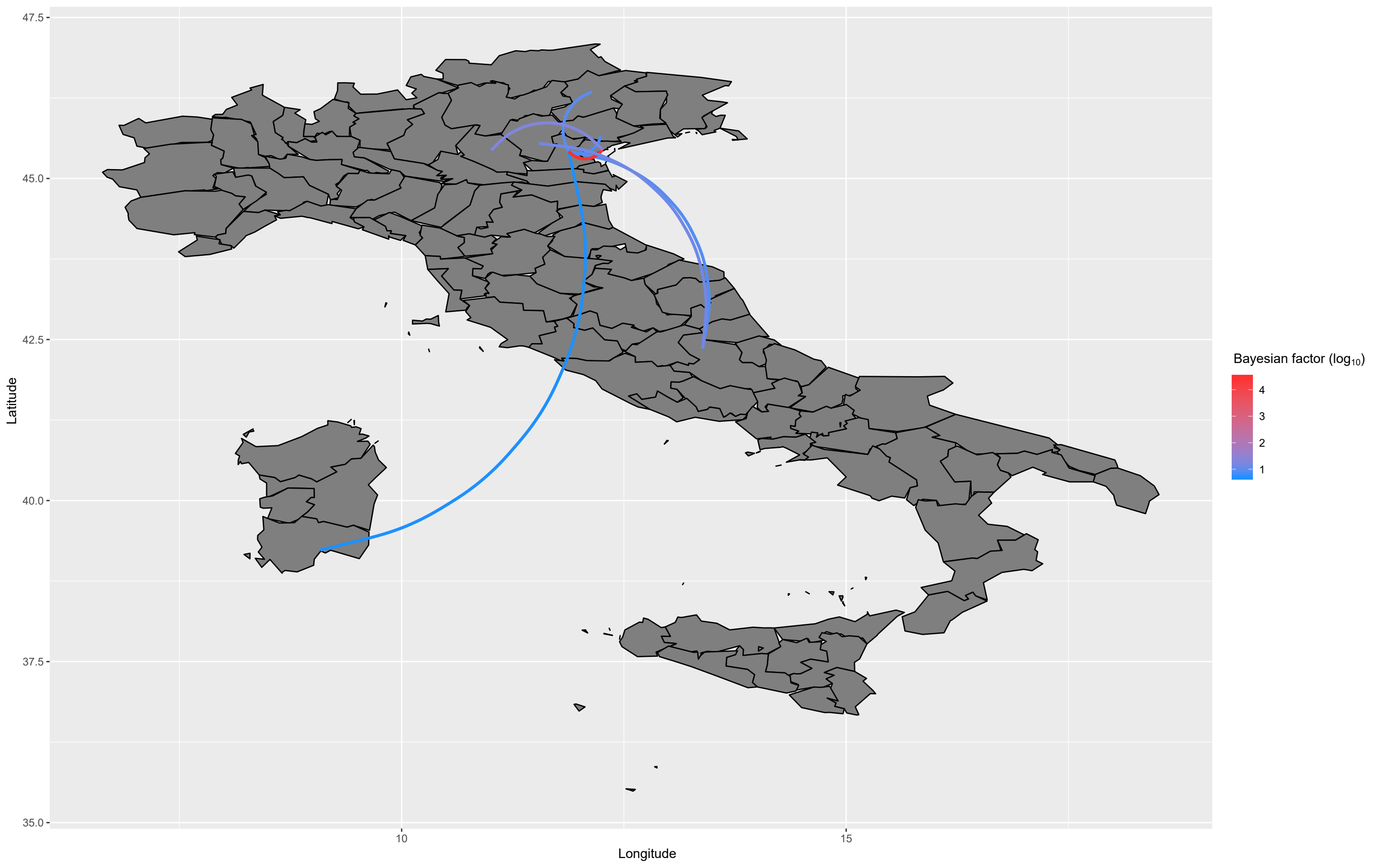

Supplement: Supplementary file 1 [file viruses-13-01033-s001.zip › viruses-1191040-supplementary/Supplementary figure 2.pdf]
